# A simplified non-coplanar volumetric modulated arc therapy for the whole brain radiotherapy with hippocampus avoidance

**DOI:** 10.3389/fonc.2023.1143564

**Published:** 2023-04-17

**Authors:** Juan Xue, Sunian Jin, Hongtao Zhang, Kun Zou, Junxiu Sheng, Jinhai Tang, Wanying Zhao, Ping Yang, Lufan Tang, Xiupeng Lv, Li Lv

**Affiliations:** ^1^ The Department of Radiation Oncology, First Affiliated Hospital, Dalian Medical University, Dalian, Liaoning, China; ^2^ The Department of Pathology, Second Affiliated Hospital, Dalian Medical University, Dalian, Liaoning, China

**Keywords:** hippocampal avoiding, whole brain radiation therapy, brain metastases, IMRT, VMAT, coplanar, noncoplanar

## Abstract

**Purpose:**

To evaluate the feasibility of using a simplified non-coplanar volumetric modulated arc therapy (NC-VMAT) and investigate its dosimetric advantages compared with intensity modulated radiation therapy (IMRT) and coplanar volumetric modulated arc therapy (C-VMAT) for hippocampal-avoidance whole brain radiation therapy (HA-WBRT).

**Methods:**

Ten patients with brain metastase (BM) were included for HA-WBRT. Three treatment plans were generated for each case using IMRT, C-VMAT, and NC-VMAT, respectively.

**Results:**

The dosimetric results of the three techniques complied roughly with the RTOG 0933 criteria. After dose normalization, the V_30Gy_ of whole brain planned target volume (WB-PTV) in all the plans was controlled at 95%. Homogeneity index (HI) of WB-PTV was significantly reduced in NC-VMAT (0.249 ± 0.017) over IMRT (0.265 ± 0.020, *p*=0.005) and C-VMAT (0.261 ± 0.014, *p*=0.020). In terms of conformity index (CI), NC-VMAT could provide a value of 0.821 ± 0.010, which was significantly superior to IMRT (0.788 ± 0.019, *p*<0.001). According to D_2%_ of WB-PTV, NC-VMAT could provide a value of 35.62 ± 0.37Gy, significantly superior to IMRT (36.43 ± 0.65Gy, *p*<0.001). According to D_50%_ of WB-PTV, NC-VMAT can achieve the lowest value of 33.18 ± 0.29Gy, significantly different from IMRT (33.47 ± 0.43, *p*=0.034) and C-VMAT (33.58 ± 0.37, *p*=0.006). Regarding D_2%_, D_98%_, and D_mean_ of hippocampus, NC-VMAT could control them at 15.57 ± 0.18Gy, 8.37 ± 0.26Gy and 11.71 ± 0.48Gy, respectively. D_2%_ and D_mean_ of hippocampus for NC-VMAT was significantly lower than IMRT (D_2%_: 16.07 ± 0.29Gy, *p*=0.001 D_mean_: 12.18 ± 0.33Gy, *p*<0.001) and C-VMAT (D_2%_: 15.92 ± 0.37Gy, *p*=0.009 D_mean_: 12.21 ± 0.54Gy, *p*<0.001). For other organs-at-risk (OARs), according to D_2%_ of the right optic nerves and the right lenses, NC-VMAT had the lowest values of 31.86 ± 1.11Gy and 7.15 ± 0.31Gy, respectively, which were statistically different from the other two techniques. For other organs including eyes and optic chiasm, NC-VMAT could achieve the lowest doses, different from IMRT statistically.

**Conclusion:**

The dosimetry of the three techniques for HA-WBRT could roughly comply with the proposals from RTOG 0933. After dose normalization (D_95%_=30Gy), NC-VMAT could significantly improve dose homogeneity and reduce the D_50%_ in the brain. Besides, it can reduce the D_2%_ of the hippocampus, optic nerves, and lens. With this approach, an efficient and straightforward plan was accomplished.

## 1 Introduction

BM is the most common intracranial tumor in adults. It is estimated that about 20% of patients diagnosed with cancer will develop BM (1–3), and the incidence of BM is ten times higher than that of primary malignancies (4). Although the survival time of patients with BM treated only with hormone and other symptomatic treatments is 1-2 months (5), the median survival time can be extended to 4-6 months after WBRT (6). WBRT has become an important treatment option for patients with multiple BM due to the limited penetration of systemic chemotherapy on the blood-brain barrier (BBB) (7) and the control effect of WBRT on BM.

However, the toxic side effects of radiation therapy on the central nervous system in long-term survivors with BM who have previously received WBRT, have attracted more and more attention. About 50-90% of patients who have received WBRT show progressive cognitive dysfunction (8). The pathophysiological mechanism of radiation-induced cognitive decline is complex (9–11). Relevant studies have shown that radiation-induced hippocampal injury plays an essential role in the decrease of neurocognitive function in patients after intracranial irradiation, especially in the decrease of learning, memory, and spatial processing functions (12). However, other studies (13) have shown that memory function is not related to the whole hippocampal structure but to pyramidal cells and granule cells in the dentate gyrus of the hippocampus. The inflammatory response in the proliferation zone of neural stem cells in the subgranular layer of the hippocampus may be one of the mechanisms of neurocognitive decline after cranial irradiation.

Previous published single-arm phase II clinical study of Gondi et al. (14)avoided the hippocampal neural stem cell region using IMRT techniques, and it has indicated that D_100%_ of the hippocampus oversteps 9Gy and D_max_ of the hippocampus oversteps 16Gy with the prescription dose of 30Gy in 10 fractions in HA-WBRT were connected with memory impairments. Additionally, Paul D et al. (15) conducted a phase III clinical trial and the results showed that compared with WBRT plus memantine, HA-WBRT plus memantine effectively spares the hippocampal neuro regenerative niche to better preserve cognitive function with the same hippocampal protective dose as Gondi's study.

HA-WBRT requires the PTV to obtain a sufficient prescription dose while minimizing the irradiation dose to the hippocampal area. Due to its irregular shape and deep location in the brain, protecting the hippocampal poses a significant challenge to implementing radiotherapy planning. With the emergence of precision radiotherapy, IMRT, VMAT, and TOMO are the most commonly used techniques for HA-WBRT. HA-WBRT was performed by Gondi et al. (16) using TOMO and linear accelerator-based IMRT techniques, and they found that both methods could achieve acceptable coverage and homogeneity and TOMO technology has more advantages in protecting the hippocampus. One of the characteristics of TOMO is that it can rapidly achieve dose drop in a small space. However, the equipment cost of TOMO is high, which is not affordable for many small-scale hospitals. In the study of Wang et al. (17), they achieved HA-WBRT by using IMRT and found that the treatment time was 576.6s, almost seven times than 3D-CRT. The drawn-out time of treatment was regarded as one of the disadvantages of complex beam IMRT. It takes a long time for patients to be treated on the treatment couch, which may cause patients discomposure and provide the opportunity for tumor movements. In 2007, a novel form of arc therapy called VMAT was introduced (18). VMAT delivers a highly conformal radiation dose to the target by simultaneously modulating gantry rotation, dose rate, and MLC position (19). Despite delivering plenty of monitor units, the possible superiority of VMAT over IMRT consists of the capability of cutting down the total number of Mus, and it can reduce delivery times, which probably lessens the leak radiation dose of patients. Dosimetry of dual-arc coplanar conventional VMAT for HA-WBRT has been showed in earlier researches following RTOG 0933 recommendations (20–22), and it has advantages in some dosimetric aspects. However, the large field of conventional coplanar dual-arc VMAT for WB-PTV demanded a wide jaw opening, and it can contribute to limited multi-leaf collimator movements, which has been stated in earlier study (23). In the worst case, the multi-leaf collimator could not shelter the OARs in the distal portion of PTV. Some studies (24) have designed NC-VMAT technology and have achieved dosimetric advantages. However, the technique is more complex and challenging for physicists, and multi-arc application also increases the treatment time. Herein, we present a new treatment technology of NC-VMAT generated only using one single coplanar arc and one non-coplanar arc, and evaluate the dosimetric performance of HA-WBRT using IMRT, C-VMAT, and NC-VMAT. This is the first reported experience of dosimetric comparison among the three techniques for HA-WBRT.

## 2 Materials and methods

### 2.1 Patient selection and CT simulation

10 patients with BM were contained in this retrospective planning research. All patients had been diagnosed with cerebral metastasis and had a previous diagnosis of the primary tumor. CT images were attained by a Philips large Bore CT simulator. CT scans with a slice thickness of 1.5 mm including the entire head region. All patients wore a thermoplastic mask which immobilized the patient’s head and were in a supine position during CT simulation.

### 2.2 Hippocampal, target and other OARs contouring

The enhanced 3D brain MRI axial T1-weighted sequences and T2-flair sequences which were the most recent from simulation were obtained and a slice thickness for MRI scans was 1.5mm. The CT and MRI data were transmitted to Elekta Monaco Version 5.11.02 treatment planning system (TPS). Each structure was contoured by a practiced radiation oncologist on the CT images refer to the fused MRI images on Monaco Version 5.11.02 TPS. The hippocampus can be accurately delineated according to RTOG 0933. Establish a hippocampal avoidance zone (HAZ) at a 5mm uniform margin around the paired hippocampus to achieve the dose drop between the hippocampus and the planning target volume. WB-PTV was defined as the remaining part of the whole brain parenchyma excluding the HAZ. Other structures of OARs which need to be contoured include brain, brain stem, eyes, lenses, optic nerves and optic chiasm.

### 2.3 Dose prescription and three treatment plan techniques

All planned treatment prescriptions were 30Gy in 10 fractions to the WB-PTV, and all the plans were initially normalized so that 100% of the prescription was delivered to 95% of the PTV (D_95%_) according to ICRU published Report 83 (25, 26). The acceptable compliance criteria for the planning doses of the WB-PTV and OARs according to the RTOG 0933 were shown in **Table 1**. For each patient, three treatment plans (IMRT, C-VMAT, and NC-VMAT) were optimized with 6MV photon beams for a linear accelerator (Axesse, Elekta Medical Systems) equipped with 80 pairs of the multi-leaf collimator (5-mm leaf width at isocenter) with a maximum dose rate of 600MU/min. All three plans were optimized using Monaco version 5.11.02 TPS. The dose calculation used the photon Monte Carlo calculation algorithm and the calculation grid was set at an initial value of 1.5mm. Three types of plans were generated: one with 9-field non-coplanar IMRT using previously reported beam arrangement (16), another is two coplanar full VMAT arcs and the last is composed of one 360°coplanar VMAT arc and one non-coplanar VMAT arc with arc ranging from 182° to 310° moving clockwise with couch at 90°. The specific beam arrangements of the three plans are shown in **Table 2**.

**Table 1:** Dose criteria of RTOG 0933 protocol.

	Dosimetry Metric	Per protocol	Acceptable variation
WB-PTV	D_2%_ (Gy)	≤37.5	37.5 to 40
	D_98%_ (Gy)	≥25	22.5 to 25
	V_30Gy_ (100%)	≥95	90 to 95
Hippocampus	D_100%_ (Gy)	≤9	9 to 10
	D_max_ (Gy)	≤16	16 to 17
Optic nerves	D_max_ (Gy)	≤30	30 to 37.5
Optic chiasm	D_max_ (Gy)	≤30	30 to 37.5

WB-PTV, whole brain planned target volume.

**Table 2:** 9-Field IMRT, C-VMAT and NC- VMAT beam arrangements.

	Couch	Gantry	Collimator
(A)9-Field IMRT beam arrangement			
Beam 1	320	30	40
Beam 2	330	310	340
Beam 3	45	180	90
Beam 4	10	104	25
Beam 5	16	49	28
Beam 6	276	9	0
Beam 7	330	265	350
Beam 8	16	317	340
Beam 9	270	319	90
(B)C-VMAT beam arrangement			
Arc 1	0	CW 182-178	45
Arc 2	0	CCW178-182	135
(C)NC-VMAT beam arrangement			
Arc 1	0	CW 182-178	45
Arc 2	90	CW 182-310	0

a. IMRT, intensity-modulated radiation therapy b. C-VMAT, coplanar volumetric modulated arc therapy c. NC-VMAT, non-coplanar volumetric modulated arc therapy d. CW, clockwise e. CCW, counter-clockwise. The full names of IMRT, C-VMAT and NC-VMAT are no longer marked in all subsequent tables and figures.

### 2.4 Planning evaluation

1. Compliance criteria and critical structure constraints in RTOG 0933 were used to evaluate the plans and see **Table 1** for details.

2. The dose-volume histogram (DVH) of the WB-PTV, hippocampus, and other OARs in three plans were generated on Monaco TPS.

3. For each patient, D_2%_, D_98%_, D_50%_, CI, and HI for WB-PTV were evaluated:

a. D_2%_: the greatest dose delivered to 2% of the WB-PTV.

b. D_98%_: the dose delivered to 98% of the WB-PTV.

c. D_50%_: the dose delivered to 50% of the WB-PTV.

d. CI: the CI (27) was defined as:


$$CI=\frac{V_{Tpres}^{2}}{TV\times V_{pres}}$$

V_Tpres_ is defined as the volume within the target receiving a dose at least reaching

the prescription dose. V_pres_ is defined as the volume receiving a dose at least reaching the prescription dose. TV represents the target volume. The value of CI ranges from 0 to 1, and larger CI values closer to 1 indicate superior conformity.

e. HI: the HI (28) was defined as:


$$HI=\frac{D_{2\%}-D_{98\%}}{D_{median}}$$

HI represents the dose homogeneity of the target volume. D_median_ represents the median dose of the target volume. HI values closer to 0 represent better homogeneity.

4. The dose parameters for OARs used as followed:

a. D_max_, D_100%_, D_98%_, D_2%_ and D_mean_ of hippocampal.

b. D_2%_ of eyes.

c. D_2%_ of lens.

d. D_2%_ of optic nerves.

e. D_2%_ of optic chiasm.

### 2.5 Statistical analysis

One-way Analysis of Variance (ANOVA) and the Least Significant Distance (LSD) *post-hoc* tests were used for analyzing dosimetric parameters among the three treatment plans on IBM SPSS Version 25.0 statistical software. *p* values of<0.05 were regarded to indicate a statistically significant difference.

### 2.6 Quality assurance

We have performed quality assurance (QA) procedures for all the plans to validate the delivery feasibility of IMRT, C-VMAT and NC-VMAT treatment plans. All the plans were delivered on a linear accelerator (Axesse, Elekta Medical Systems) and were measured by ArcCHECK diode array. After finishing the measurements, we assessed the consistency between the original dose distribution and the measured doses by using Gamma analysis with two groups of dose difference/distance criteria (3%/3 mm and 2%/2 mm) on SunCHECK software.

## 3 Results

### 3.1 Planning template

A template of NC-VMAT was generated on Monaco TPS to standardize plan starting points and contribute to the reproducibility of results across patients. We generated a suit of regulated cost functions in each template, including Maximum, Quadratic Overdose, Target Penalty, Underdose DVH and Root Mean Square (**Table 3**).

**Table 3:** Cost function template for planning a NC-VMAT of HA-WBRT plan in Monaco.

	Cost Function	Reference Dose(Gy)	Isoeffect
Hippocampus-L	Max	8	Over all voxels
	QO	9	RMS=50
			Over all voxels
Hippocampus-R	Max	8	Over all voxels
	QO	9	RMS=50
			Over all voxels
WB-PTV	Target Penalty	30	Minimum Volume=90%
	Underdose DVH	30	Minimum Volume=95%
Lens-L	Max	8	Over all voxels
Lens-R	Max	8	Over all voxels
Optic chiasm	QO	32	RMS=100
			Over all voxels
Optic nerve-L	QO	32	RMS=100
			Over all voxels
Optic nerve-R	QO	32	RMS=100
			Over all voxels
Body	QO	30	RMS=100
			0.5 Shrink
	Max	3600	Over all voxels
	QO	2000	RMS=100
			1 Shrink

a HA-WBRT, Hippocampal-avoidance whole brain radiation therapy b. QO, quadratic overdose c. RMS, root mean square.

### 3.2 The dosimetry and dose distribution of WB-PTV

**Figure 1:**
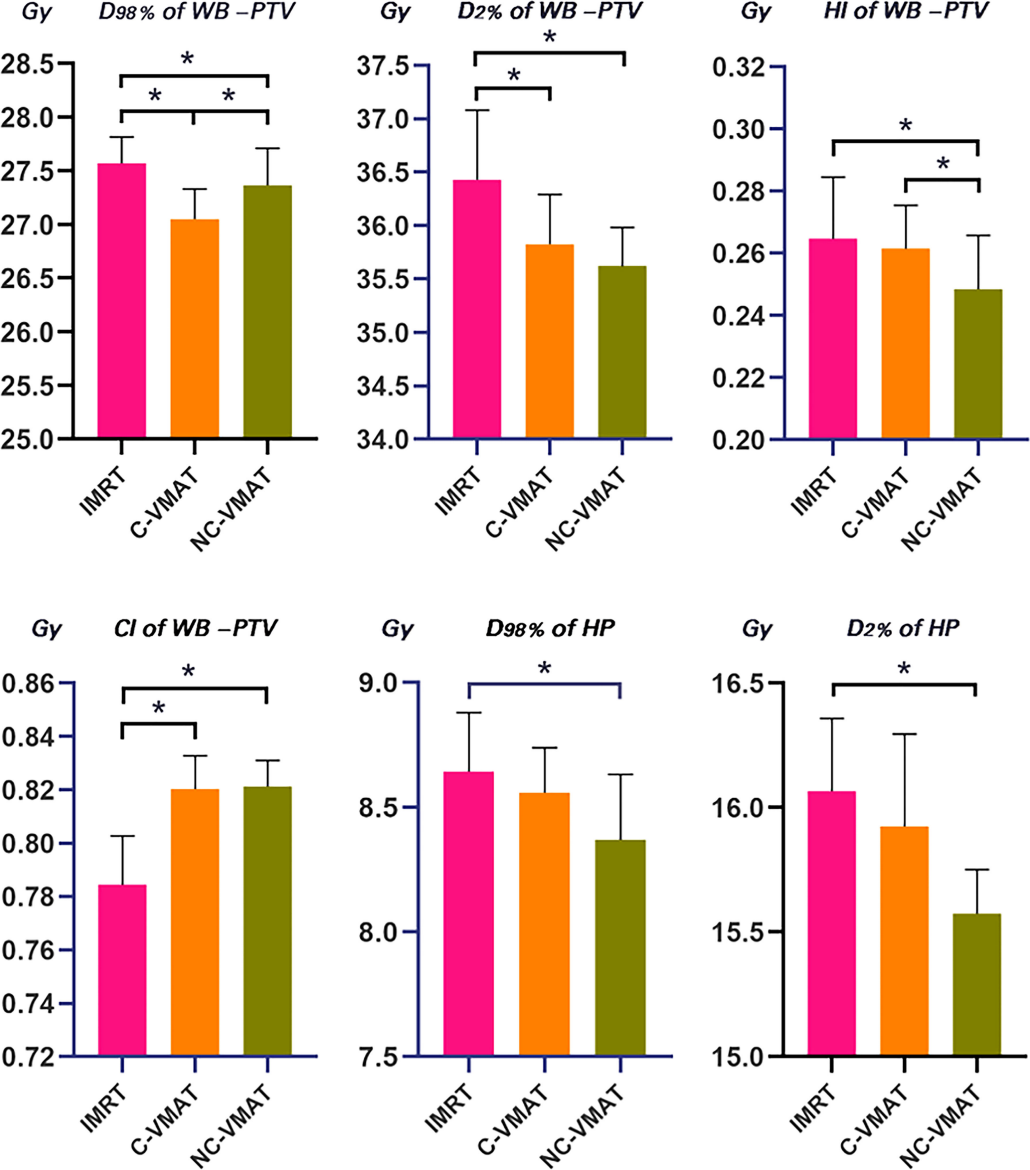
Comparison for mean values of D_98%_, D_2%_, HI and CI of WB-PTV and D_2%_ and D_98%_ of hippocampus for IMRT, C-VMAT and NC-VMAT plans. a. *p<0.05 (one-way ANOVA, LSD *post-hoc* test) b. HP, hippocampus c. Error bars was caculated by standard deviation (SD).

**Figure 2:**
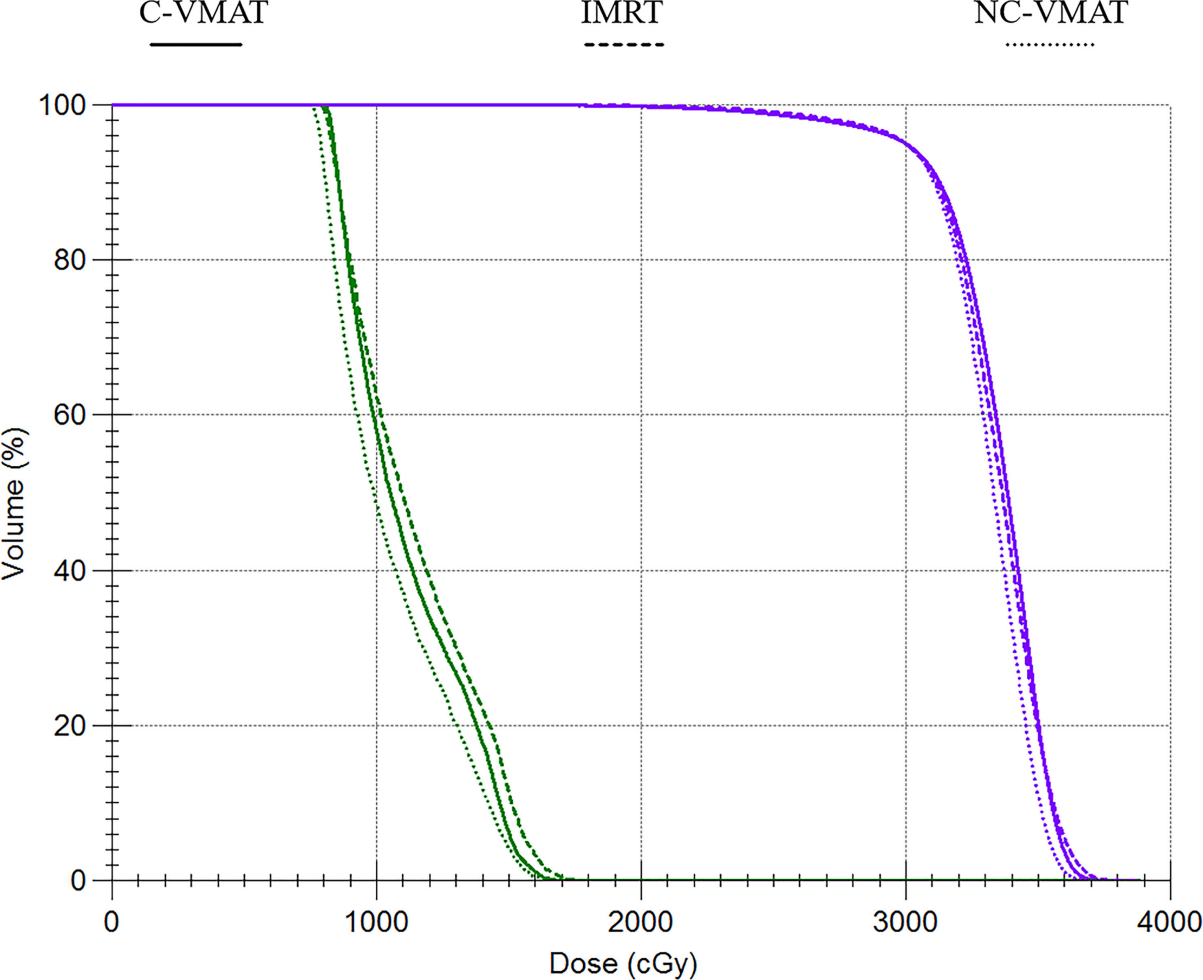
Dose-volume histograms (DVHs) comparison for WB-PTV and OARs between IMRT, C-VMAT and NC-VMAT plans. a. The purple line represents the WB-PTV b. Green line represents the hippocampus.

**Figure 3:**
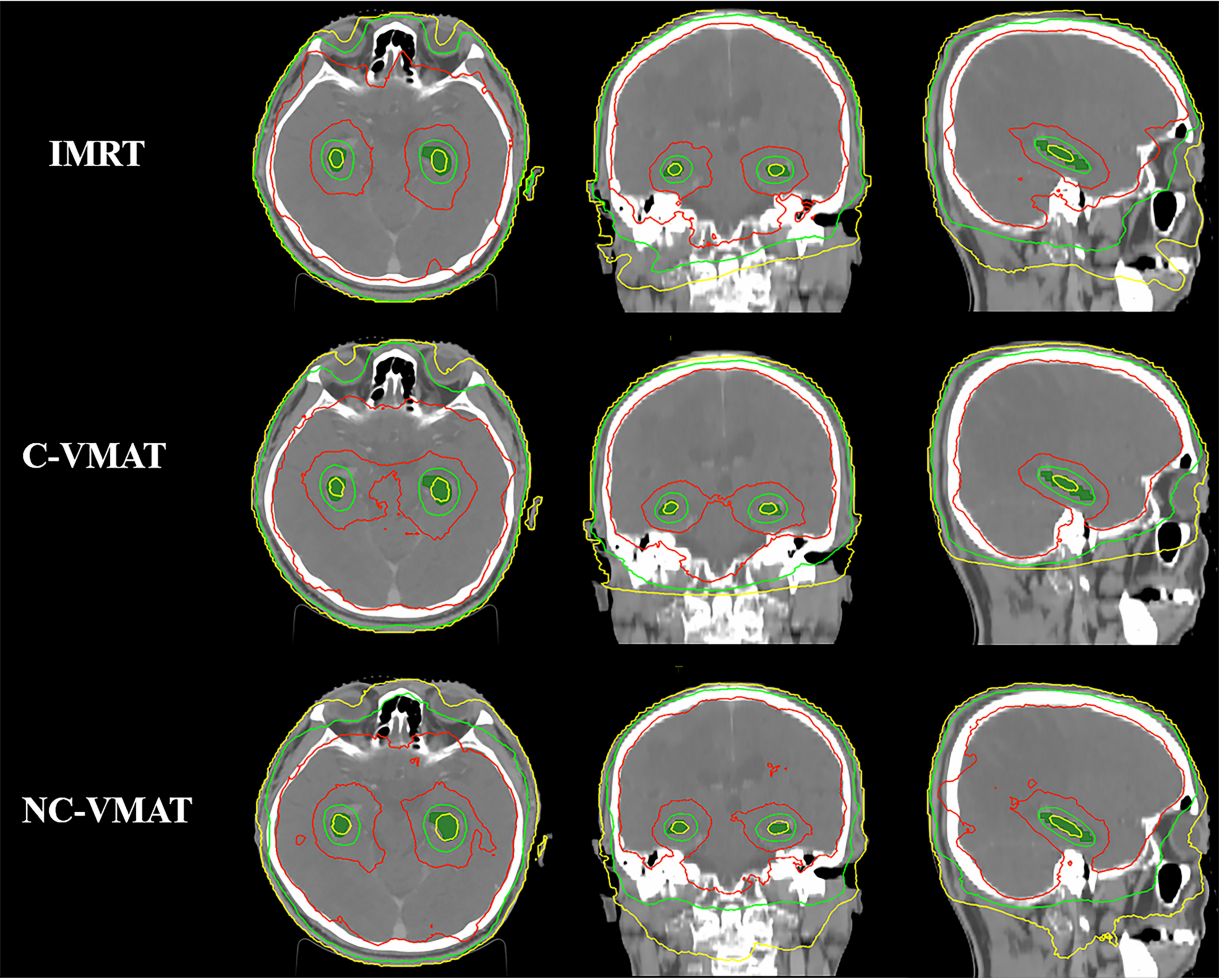
Comparison of some special isodose lines distribution between IMRT, C-VMAT and NC-VMAT plans. a. The red line represents the isodose line of 30Gy b. The green line represents the isodose line of 17Gy c. The yellow line represents the isodose line of 9Gy.

**Table 4** presented the mean dosimetric values as mean values ± standard deviation (SD) of WB-PTV for the ten patient datasets and the pairwise comparisons between IMRT, C-VMAT and NC-VMAT. The doses of the structures in the three plans roughly complied with the RTOG 0933 standard. After dose normalization, the V_30Gy_ of whole brain planned target volume (WB-PTV) in all the treatment plans was controlled at 95%. In the evaluation of hotspots, the mean D_2%_ for NC-VMAT was the lowest in the three modalities, and it showed a significantly lower D_2%_ compared to IMRT (35.62 ± 0.37Gy *vs*. 36.43 ± 0.65Gy, *p*<0.001). In terms of the coldspots, the mean D_98%_ for C-VMAT was the highest and significantly higher compared with NC-VMAT (27.05 ± 0.28Gy *vs*. 27.37 ± 0.34Gy, *p*=0.003) and IMRT (27.05 ± 0.28Gy *vs*. 27.57 ± 0.25Gy, *p*<0.001). In terms of the D_50%_, NC-VMAT was lower than both IMRT (33.18 ± 0.29Gy *vs*. 33.47 ± 0.43Gy, *p*=0.034) and C-VMAT (33.18 ± 0.29Gy *vs*. 33.58 ± 0.37Gy, *p*=0.006) and comparable to them. In addition, NC-VMAT provided a mean HI of 0.249 ± 0.017, lower significantly than C-VMAT (0.261 ± 0.014, *p*=0.020) and IMRT (0.265 ± 0.020, *p*=0.005). In terms of CI, both NC-VMAT (0.821 ± 0.010, *p*<0.001) and C-VMAT (0.820 ± 0.012, *p*<0.001) were comparable to IMRT (0.788 ± 0.019), and there is no statistically significant difference which was found between NC-VMAT and C-VMAT (*p=*0.872). These findings indicated that the optimal dose homogeneity of the target could be achieved by NC-VMAT without reducing the prescription dose coverage, and it can provide excellent target dose conformity and reduce the D_50%_ simultaneously. Mean D_98%_, D_2%_, HI and CI of WB-PTV and their significant difference in pairwise comparisons in three plans were shown in **Figure 1**. **Figure 2** presented dose volume histograms (DVHs) of the hippocampus and WB-PTV in the three techniques for one representative patient. Some special isodose lines distribution images for one representative patient in the three techniques were shown in **Figure 3**.

**Table 4:** The dosimetric values (mean ± SD) of WB-PTV under three types of treatments (IMRT, C-VMAT and NC-VMAT) and comparison of the three treatments in terms of dosimetry metric and related *p* values.

Dosimetry Metric	IMRT	C-VMAT	NC-VMAT	*P*1	*P*2	*P*3
D_98%_ (Gy)	27.57 ± 0.25	27.05 ± 0.28	27.37 ± 0.34	<0.001*	0.039*	0.003*
D_2%_ (Gy)	36.43 ± 0.65	35.83 ± 0.46	35.62 ± 0.37	0.005*	<0.001*	0.271
D_50%_ (Gy)	33.47 ± 0.43	33.58 ± 0.37	33.18 ± 0.29	0.422	0.034*	0.006*
HI	0.265 ± 0.020	0.261 ± 0.014	0.249 ± 0.017	0.533	0.005*	0.020*
CI	0.788 ± 0.019	0.820 ± 0.012	0.821 ± 0.010	<0.001*	<0.001*	0.872

a. P1, p value between IMRT and C-VMAT b. P2, p value between C-VMAT and NC-VMAT c. P3, p value between IMRT and NC-VMAT d. *p<0.05(one-way ANOVA, LSD post-hoc test).

### 3.3 Dosimetry of hippocampus

D_98%_ and D_2%_ of the hippocampus in the three plans for each case were shown in **Table 5**. In terms of the dosimetry of the hippocampus, the mean D_98%_ in NC-VMAT, C-VMAT and IMRT were 8.37 ± 0.26Gy, 8.56 ± 0.18Gy and 8.64 ± 0.24Gy, respectively, and D_98%_ of NC-VMAT was significantly reduced compared with IMRT (8.37 ± 0.26Gy *vs*. 8.64 ± 0.24Gy, *p*<0.001). D_2%_ was the lowest for NC-VMAT (15.57 ± 0.18Gy), followed by C-VMAT (15.92 ± 0.37Gy) and IMRT (16.07 ± 0.29Gy), and D_2%_ of NC-VMAT was significantly lower compared with IMRT (*p*<0.001) and C-VMAT (*p*=0.009). According to D_mean_ of the hippocampus, NC-VMAT can provide the lowest value of 11.71 ± 0.48Gy, which is significantly lower than IMRT (12.18 ± 0.33Gy, *p*<0.001) and C-VMAT (12.21 ± 0.54Gy, *p*<0.001). The details are shown in **Table 6**. Mean D_2%_ and D_98%_ of the hippocampus and their significant difference in pairwise comparisons in three plans were shown in **Figure 1**.

**Table 5:** The dosimetric values of hippocampus under three types of treatments for each case (IMRT, C-VMAT and NC-VMAT).

	IMRT		C-VMAT		NC-VMAT	
	D_98%_(Gy)	D_2%_(Gy)	D_98%_(Gy)	D_2%_(Gy)	D_98%_(Gy)	D_2%_(Gy)
PT1	8.76	16.31	8.83	16.33	8.56	15.81
PT2	8.89	15.83	8.52	15.43	8.59	15.53
PT3	8.76	16.57	8.43	15.61	8.39	15.69
PT4	8.65	15.85	8.62	16.40	8.44	15.70
PT5	8.74	15.60	8.80	15.75	8.54	15.51
PT6	8.96	16.32	8.68	15.72	8.59	15.71
PT7	8.15	16.25	8.25	15.73	7.75	15.42
PT8	8.55	15.97	8.39	15.69	8.14	15.20
PT9	8.45	15.92	8.54	16.49	8.26	15.66
PT10	8.52	16.05	8.52	16.09	8.43	15.50
Avg.	8.64	16.06	8.56	15.93	8.37	15.57
STD.	0.24	0.29	0.18	0.37	0.26	0.18

a. Avg., average b. STD., standard deviation.

**Table 6:** The dosimetric values (mean ± SD) of hippocampus under three types of treatments (IMRT, C-VMAT and NC-VMAT) and comparison of the three treatments in terms of dosimetry metric and related *p* values.

Dosimetry Metric	IMRT	C-VMAT	NC-VMAT	*P*1	*P*2	*P*3
D_100%_ (Gy)	8.32 ± 0.32	8.13 ± 0.21	8.03 ± 0.24	0.002*	<0.001*	0.078
D_max_ (Gy)	17.34 ± 0.57	17.13 ± 0.46	16.81 ± 0.41	0.254	0.008*	0.092
D_98%_ (Gy)	8.64 ± 0.24	8.56 ± 0.18	8.37 ± 0.26	0.099	<0.001*	0.099
D_2%_ (Gy)	16.07 ± 0.29	15.92 ± 0.37	15.57 ± 0.18	0.252	0.001*	0.009*
D_mean_(Gy)	12.18 ± 0.33	12.21 ± 0.54	11.71 ± 0.48	0.739	<0.001*	<0.001*

a. P1, p value between IMRT and C-VMAT b. P2, p value between C-VMAT and NC-VMAT c. P3, p value between IMRT and NC-VMAT d. *p<0.05(one-way ANOVA, LSD post-hoc test).

### 3.4 Dosimetry of optic chiasm, optic nerves, eyes and lenses

**Table 7** presented the mean dosimetric values as mean values ± standard deviation (SD) of other OARs for the ten patient datasets and the pairwise comparisons between IMRT, C-VMAT and NC-VMAT. In terms of the right lenses, the mean D_2%_ for NC-VMAT was significantly superior to C-VMAT (7.15 ± 0.31Gy *vs*. 7.43 ± 0.29Gy, *p*=0.018) and IMRT (7.15 ± 0.31Gy *vs*. 7.42 ± 0.31Gy, *p*=0.022). In terms of the left lens, the mean D_2%_ for NC-VMAT was significantly lower than C-VMAT (7.18 ± 0.22Gy *vs*. 7.44 ± 0.41Gy, *p*=0.045), but there was no significant difference between NC-VMAT and IMRT (7.18 ± 0.22Gy *vs*. 7.38 ± 0.18Gy, *p*=0.646). According to the right optic nerves, NC-VMAT had the lowest values of 31.86 ± 1.11Gy, which were statistically different from IMRT (33.93 ± 0.45Gy, *p*<0.001) and C-VMAT (33.02 ± 0.88Gy, *p*=0.011). For other organs including eyes and optic chiasm, NC-VMAT could achieve the lowest doses, and the difference was statistically significant only compared with IMRT.

**Table 7:** The dosimetric values (mean ± SD) of other OARs under three types of treatments (IMRT, C-VMAT and NC-VMAT) and comparison of the three treatments in terms of dosimetry metric and related *p* values.

Structure	Dosimetry Metric	IMRT	C-VMAT	NC-VMAT	*P*1	*P*2	*P*3
Optic nerves-L	D_2%_(Gy)	32.13 ± 1.18	32.79 ± 1.24	32.14 ± 0.91	0.170	0.983	0.176
Optic nerves-R	D_2%_(Gy)	33.93 ± 0.45	33.02 ± 0.88	31.86 ± 1.11	0.040*	<0.001*	0.011*
Optic chiasm	D_2%_(Gy)	34.74 ± 1.21	34.39 ± 0.84	33.86 ± 0.59	0.378	0.034*	0.183
Lens-L	D_2%_(Gy)	7.38 ± 0.18	7.44 ± 0.41	7.18 ± 0.22	0.646	0.109	0.045*
Lens-R	D_2%_(Gy)	7.42 ± 0.31	7.43 ± 0.29	7.15 ± 0.31	0.913	0.022*	0.018*
Eye-L	D_2%_(Gy)	20.05 ± 1.59	18.26 ± 2.29	18.60 ± 2.60	0.003*	0.011*	0.515
Eye-R	D_2%_(Gy)	23.18 ± 2.38	19.02 ± 1.26	18.36 ± 2.70	<0.001*	<0.001*	0.387

a. P1, p value between IMRT and C-VMAT b. P2, p value between C-VMAT and NC-VMAT c. P3, p value between IMRT and NC-VMAT d. *p<0.05(one-way ANOVA, LSD post-hoc test).

### 3.5 Quality assurance

**Figure 4:**
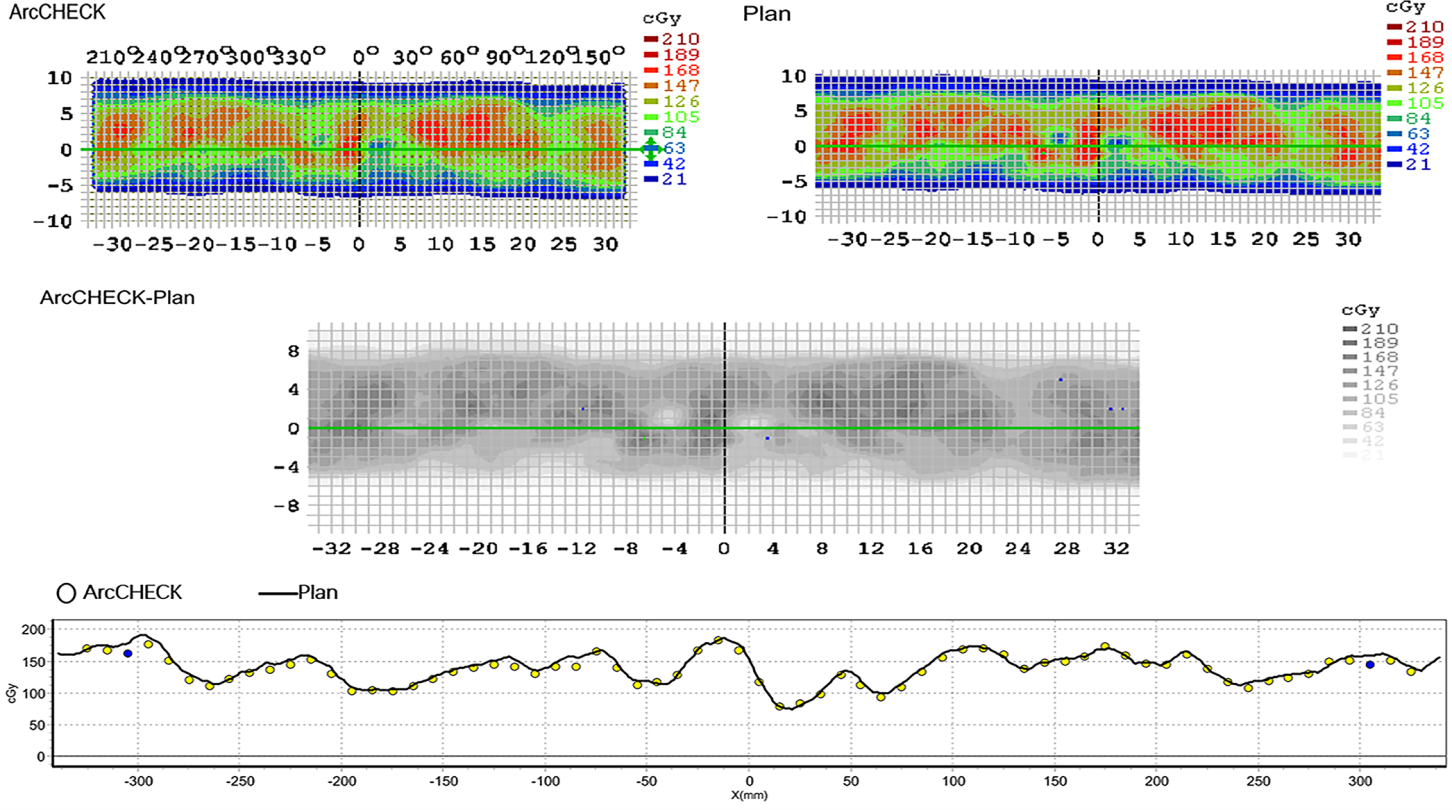
The agreement between the measured dose distribution and the original dose distribution for NC-VMAT of one representative patient. a. ArcCHECK: the measured dose distribution b. Plan: the original dose distribution.

As shown in **Table 8** and **Figure 4**, the measured dose distributions had good agreement with the original doses for all the cases. We conclude that all the plans achieved good plan quality and were clinically feasible.

**Table 8:** Quality assurance.

	IMRT		C-VMAT		NC-VMAT	
	3%/3mm	3%/2mm	3%/3mm	3%/2mm	3%/3mm	3%/2mm
PT1	99.7	99.2	99.3	97.0	99.2	97.0
PT2	97.9	94.1	99.4	97.3	97.3	96.7
PT3	98.1	95.6	98.9	96.9	97.2	95.9
PT4	98.3	96.1	99.2	97.5	99.3	96.2
PT5	97.6	94.6	98.4	98.8	98.1	97.3
PT6	99.6	95.3	96.8	98.1	99.0	96.9
PT7	97.1	97.2	98.3	97.4	97.4	98.0
PT8	99.4	96.9	99.1	98.0	98.6	96.4
PT9	98.4	97.1	98.6	96.5	97.6	97.2
PT10	97.2	95.9	97.8	96.8	99.3	96.8
Avg.	98.3	96.2	98.6	97.4	98.3	96.8
STD.	1.0	1.5	0.8	0.7	0.9	0.6

a. Avg., average b. STD., standard deviation.

## 4 Discussion

HA-WBRT has got a lot of attention because of its advantages of protecting the neurocognitive function and RTOG 0933 has made great progress to show an improvement in the life quality of patients. In addition, we should focus on the consideration of whether protection of the hippocampus and surrounding areas (extending the hippocampal margin by 5 mm) would lead to a theoretical probability of tumor recurrence in these areas. In the study of Vinai Gondi et al. (29), it was found that only 8.6% of the patients had metastases within the circumferential hippocampus region (extending 5 mm from the edge of the hippocampus), but none of the lesions were found within the hippocampus areas. In another study by Harth et al. (30), the risk of the occurrence of metastases in the hippocampus region for HA-WBRT had increased by only about 0.2%. In conclusion, the cognitive benefit of patients treated with HA-WBRT is much higher than the risk of intracranial metastasis in the hippocampus.

In this paper, IMRT, C-VMAT and NC-VMAT had been made a comparison in the treatment of ten individuals diagnosed with BM. All dosimetric parameters in three treatment plans could achieve the RTOG 0933 criteria (**Table 1**) nearly. Radiation doses to the hippocampus were decreased while the target coverage was not compromised. At the same time, we discovered that NC-VMAT achieved the most homogeneous dose distribution (mean HI = 0.249) and greatly reduce the D_50%_ (mean D_50%_ = 33.18Gy) of WB-PTV among the three techniques. In terms of D_2%_ and CI of WB-PTV, NC-VMAT was superior on both values compared to the other two methods, but it was statistically different from only one of the methods. Besides, we found that NC-VMAT was significantly superior to IMRT in terms of D_2%_ of the hippocampus, the right lenses and the right optic nerves. In general, NC-VMAT has obvious advantages in the dosimetry of the target and OARs.

The effort to reduce neurocognitive toxicity *via* advanced and conformal hippocampal avoidance techniques such as those achieved with helical tomotherapy, IMRT and VMAT has been studied. According to the research of Gondi et al. (14), they achieved D_max_ of 15.3Gy and D_medium_ of 7.8Gy to the hippocampus by using linac-based IMRT for HA-WBRT. The maximum dose of PTV larger than 40Gy (133% of the prescribed dose) was displayed. The plan used nine non-coplanar fields at seven different couch angles and we can deem that the treatment delivery could be time-wasting by using such a complex beam plan. Nevelsky et al. (31) used Elekta equipment and Monaco TPS with nine non-coplanar IMRT beams and only two different couch angles for the purpose of bringing down the treatment delivery time. Following the RTOG guidelines, although the study had shown superior doses of hippocampus protection, they still had long treatment times of around twelve minutes. In addition, they achieved 37.2Gy of D_2%_ to the WB-PTV, which approached to the maximal acceptable critical value of 37.5Gy nearly from the RTOG 0933 protocol and achieved a value of 0.36 of HI. In our study for NC-VMAT, the D_2%_ and the HI were 35.62Gy and 0.249, respectively, and this resulted in a decreased hot spot and an ameliorative homogeneity of dose distribution in the WB-PTV.

Using VMAT planning for HA-WBRT has been studied by several researchers (20–22, 32–34). Hsu et al. (33) used VMAT technique with a single arc for HA-WBRT with a simultaneous integrated boost. They achieved to deliver treatment in 3–4min and this is a very extraordinary result. However, they provided a value of 0.39 of mean HI, which is still barely satisfactory. Pokhrel et al. evaluated a VMAT technique using two full coplanar arcs sparing the hippocampus and other OARs. On VMAT plans, the superior mean HI of WB-PTV was 0.23, comparable to our results of 0.249 in NC-VMAT. However, the mean V_30Gy_ to WB-PTV was 90.5%, close to the minimum acceptable value in the RTOG 0933 criterion of 90%. which was lower than 95% in our NC-VMAT technique. An article by Kim et al. (34) showed that an inclined head position of around 11° in simulation produced a better dose homogeneity in the PTV and lowered doses to the hippocampi and optic apparatus than with a non-inclined head position using C-VMAT. The inclined head position provided a HI value of 0.4 for WB-PTV and our NC-VMAT technique can provide an improved HI value of 0.249. They had a poor prescription dose coverage V_30Gy_ of 90%, which was obviously inferior to ours. Besides, it achieved 10.45 Gy for hippocampi D_min,_ which is higher than 8.03Gy in our NC-VMAT technique. As a result, we can provide a superior dose homogeneity, prescription dose coverage and does of hippocampus protection without additional patient setup effort.

Adams et al. (32) suggested that the large field of WB-PTV in a conventional coplanar arc requires a wide jaw opening and it could produce a low dose to the hippocampus region because of the limited multi-leaf collimator movements. For this reason, Adams and colleagues provided a split-arc partial-field technique. This technique’s dosimetric results are explored to find out its protection capacity to hippocampus on WBRT. The results showed that split-arc partial-field VMAT (7.86Gy, 13.23Gy) had significantly lowered average D_100%_ and D_max_ of the hippocampus compared to dual-arc VMAT (9.23Gy, 16.33Gy). However, they reported a mean D_98%_ of 25.84Gy for WB-PTV, which is lower than 27.37Gy in our NC-VMAT plan, and the decrease in coldspots may lead to a reduction in the local control of the tumor. Krayenbuehl et al. (24) assessed an automated treatment planning using NC-VMAT with four arcs: two 360° coplanar arcs, and two non-coplanar arcs. They achieved a value of 33.5Gy for D_2%_ of the WB-PTV, which significantly improved. The Hippocampus D_100%_ dose was 8.1Gy, which was reduced by 10% compared with RTOG 0933 and very similar to our NC-VMAT result with a value of 8.03Gy. However, adding arcs will influence the time of treatment delivery, weakening the time-saving treatment advantage of VMAT.

Our NC-VMAT provides a simple and convenient treatment plan, including a single coplanar arc and a single non-coplanar arc, which reduces the planning burden and time of the physicist and dramatically improves the work efficiency under the premise that excellent dosimetric results such as HI, CI, D_50%_ of WB-PTV and D_2%_ of the hippocampus can be obtained. Besides, simplification of the NC-VMAT arc can observably save the time patients spend on the treatment couch, which has a passive influence on both the satisfaction of patients and the efficiency of departments. In addition, the planning target volume of each plan was normalized to D_95%_=30Gy in this study to satisfy and obtain acceptable dose coverage to the target and make the dosimetric parameters of OARs more comparable. However, this study has several limitations. First, our analysis didn’t detect the treatment time and Mus, thus, the issue of delivery capacity is still inconclusive. Second, not all our data met the RTOG criteria fully. The reason may be related to the use of the Elekta platform. Previous studies (31) suggested that some doses in VMAT plans did not comply with RTOG criteria due to the sequencer limitation in the Ekekta platform. In addition, compared with the pencil beam algorithm, Monte Carlo algorithm used in this study is more challenging to achieve the optimization goal.

It is required to kick couch during treatment due to the complexity of IMRT beams. It is worth mentioning that the accuracy of patient setup could be descended while kicking the couch (35), which could lead to the displacement of the tumor and ultimately result in the decrease of the tumor control rate. This problem can be solved in the following ways. First, the combination of CBCT and 6 degree of freedom couch. At present, CBCT is widely used in the clinical routine of radiotherapy and can effectively reduce the patient’s positioning error by comparing with the planning CT (36). However, most institutions currently focus on only three directions (X, Y and Z) and several studies have proved that rotation errors also had a more significant impact on the accuracy of patient setup (37). 6 degree of freedom couch is a facility that can be rotated in six dimensions to adjust setup errors. It can be assumed that the setup errors of 6 degree of freedom couch combined with CBCT can further be decreased when kicking the couch to adjust the radiation beam in IMRT. Second, the use of an improved firm head fixation technique. We found that the mismatch between the head and neck and the standard pillow could cause significant instability in positioning so that the patient is easily displaced during kicking couch. The improvement of fixation devices during positioning can greatly reduce the occurrence of this situation, such as the combination of head masks and shaping pads, vacuum pads, and heat reduction pillows. Lastly, the application of MRgRT. MRI is well-established for its superior soft-tissue contrast and the patient’s image can be continuously collected during the treatment (38). So MRgRT could be a modality that could provide more accurate images so that the patient can get more precise positioning.

In our future study, this simplified NC-VMAT technique will be used to study not only the protection of the hippocampus but also other organs at risk, such as the parotid glands, scalp, cochleae and auditory canals. In addition, a simultaneous integrated boost (SIB) for BM can also be delivered in conjunction with HA-WBRT.

## 5 Conclusion

In conclusion, compared with the other two techniques, our simplified NC-VMAT shows good dosimetric advantages under the premise of meeting the dose restriction of RTOG 0933, especially in target homogeneity, conformity, the median dose of the target volume and D_2%_ of the hippocampus. Therefore, NC-VMAT provides a practical clinical treatment option for HA-WBRT.

## Data availability statement

The raw data supporting the conclusions of this article will be made available by the authors, without undue reservation.

## Author contributions

XL raised the issue, directed the study, contoured the structures and supervised trials. LL advised on the project, performed data analysis, directed the study and supervised trials. JX and SJ conducted the plans, drafted the manuscript and performed data analysis, and these two authors contributed equally to the work. HZ participated in conducting the plans and data collection. KZ and JS participated in plan review and data collection. JT, WZ, PY and LT participated in data analysis and discussion of results for the project. All authors contributed to the article and approved the submitted version.
